# Editorial: Clinical application of artificial intelligence in emergency and critical care medicine, volume IV

**DOI:** 10.3389/fmed.2023.1346070

**Published:** 2024-01-09

**Authors:** Gagandeep Dhillon, Zhongheng Zhang, Harpreet Grewal, Rahul Kashyap

**Affiliations:** ^1^Department of Internal Medicine, University of Maryland Baltimore Washington Medical Center, Glen Burnie, MD, United States; ^2^Department of Emergency Medicine, Sir Run Run Shaw Hospital, Zhejiang University School of Medicine, Hangzhou, China; ^3^Department of Radiology, Florida State University College of Medicine, Pensacola, FL, United States; ^4^Division of Pulmonary and Critical Care Medicine, Mayo Clinic, Rochester, MN, United States; ^5^Department of Research, WellSpan Health, York, PA, United States

**Keywords:** artificial intelligence, prediction, critical care, machine learning, intensive care unit

Integrating artificial intelligence (AI) into the realm of emergency and critical care medicine marks a transformative stage in healthcare delivery. In Volume IV of the research compilation titled “*Clinical application of artificial intelligence in emergency and critical care medicine*,” a collection of 16 articles highlights the burgeoning intersection between advanced technology and acute medical interventions. This compilation looks at a spectrum of innovative applications, ranging from diagnostic support systems to predictive analytics, all poised to reshape the dynamics of emergency medical response and critical patient care.

Guiot et al. presented an interesting retrospective study on COVID-19 patients. Over the last few years, there have been millions of COVID-19 cases with many deaths (1). To help manage the load on radiologists, an artificial intelligence (AI) based analysis (CACOVID-CT) was implemented to evaluate the severity of the disease on the basis of CT chests performed on those patients. Progress in machine learning and artificial intelligence has led to the creation of tools that can augment the diagnostic skills of radiologists (2). The area of the lung affected by COVID-19 Affected Area (%AA) and CT severity score (total CT-SS) were quantified to help evaluate outcome and prognosis. It is interesting to note that both %AA and CT-SS had a high correlation with length of stay, risk for invasive ventilation, ICU admission, and death during hospital stay. It alleviated the workload of radiologists by measuring the severity of lung damage.

As the pandemic continued to grow, there was an increased number of COVID-19 patients with acute respiratory distress syndrome (ARDS) in the ICU (3). However, there was limited information about predictive studies of ARDS in those patients. Zhou et al. attempted to create predictive models to establish a correlation between ARDS and COVID-19. One hundred three critically ill COVID patients were included in the study, and the development of ARDS in patients admitted to ICU was the primary outcome. Based on convolutional neural network (CNN) and extreme gradient boosting (XGBoost), two predictive models were established. Out of 104, 23 (22.3%) of patients developed ARDS. In critically ill COVID-19 patients, an integrated deep-learning model can be helpful to predict ARDS.

As the healthcare industry embarks on a paradigm shift toward a more data-driven and technologically enhanced future, this volume is a comprehensive exploration of AI's profound impact in optimizing clinical decision-making, resource allocation, and patient outcomes within the high-stakes environments of emergency and critical care settings. Saqib et al. carried out a comprehensive search in PubMed, Google Scholar, PLOS One, and Scopus to develop an understanding of AI applications in critical illness in a narrative review. They concluded that it is vital to ensure that AI systems are made robust and reliable in the care of critically ill patients. Also, there should be transparent and comprehensible reasoning behind recommendations generated by AI. Quality control measures must be in place to ensure safety and effectiveness.

Nonvariceal upper gastrointestinal bleeding (NVUGIB) in patients with decompensated cirrhosis can be critically ill and has been associated with a higher rate of readmissions and mortality (4). For patients with NVUGIB, Ungureanu et al. developed an artificial neural network with mortality as the primary outcome. Over 1000 NVUGIB patients hospitalized were divided into training and testing groups in this retrospective study. Glasgow Blatchford (GBS), AIM65, and admission Rockall (Rock) are non-endoscopic risk scores used in the past. In the study, four machine learning algorithms, Quadratic Discriminant Analysis (QDA), logistic regression (LR), Linear Discriminant Analysis (LDA), and K-Nearest Neighbor (K-NN) were used with GBS, Rock, AIM65, and others. It was noted that the machine learning models had more accuracy in identifying patients with a higher mortality risk than the current risk scores. An accuracy of 98% was seen with K-NN classifier, proving that there is scope for using machine learning in NVUGIB patients to predict mortality.

In hospitals, length of stay (LOS) indicates the efficiency of management (5). Zeleke et al. attempted to compare and develop various models to estimate LOS and prolonged LOS in patients admitted through the emergency room. Six algorithms (Random Forest (RF), Support Vector Machines (SVM), Gradient Boosting (GB), AdaBoost, K-Nearest Neighbors (KNN), and logistic regression (LR) were used, and they analyzed a total of 12,858 patients. Out of them, 61% had a prolonged LOS. The models were evaluated using the Brier score with the area under the curve, sensitivity, accuracy, precision, specificity, and F1 score.GB algorithm best predicted the accuracy of prolonged LOS, and there was tremendous potential seen in the machine learning-based methods to assess for LOS. They also give insights to help understand the risks behind increased LOS. If combined with provider expertise, they can be used to make informed decisions.

In conclusion, examining “*Clinical application of artificial intelligence in emergency and critical care medicine*,” Volume IV, has provided a nuanced insight into the transformative potential of artificial intelligence within the critical domains of emergency and critical care medicine. It encapsulates a diverse array of AI applications, ranging from real-time diagnostics to prognostic modeling, each contributing to an evolving landscape where technology complements and enhances the capabilities of healthcare professionals. Although most topics have been covered in the collection of articles, we should be cognizant of the fact that technological advancement with AI does bring to light an acute need to address ethical considerations. Healthcare industry needs to protect the values of medicine and follow fundamental ethical principles. The future calls for papers for this special topic may consider including it. As we move forward, a collaborative effort between clinicians, technologists, and policymakers is crucial to harness the full potential of artificial intelligence for improving patient care in these critical settings.

## Author contributions

GD: Conceptualization, Methodology, Visualization, Writing – original draft. ZZ: Conceptualization, Supervision, Writing – review & editing. HG: Conceptualization, Methodology, Visualization, Writing – review & editing. RK: Conceptualization, Project administration, Resources, Supervision, Writing – review & editing.
